# Caring at the transition: success and financial barriers of fidaxomicin discharge prescriptions for inpatients with *Clostridioides difficile infection*

**DOI:** 10.1017/ash.2024.484

**Published:** 2025-01-27

**Authors:** Ellen Earle, Jigar Mehta, Alexis Blecher, Matthew Shou Lun Lee

**Affiliations:** 1 Division of Infectious Diseases, Oregon Health & Science University, Portland, OR, USA; 2 Department of Pharmacy, Beth Israel Deaconess Medical Center, Boston, MA, USA; 3 Division of Infection Control/Hospital Epidemiology, Beth Israel Deaconess Medical Center, Boston, MA, USA; 4 Division of Infectious Diseases, Beth Israel Deaconess Medical Center and Harvard Medical School, Boston, MA, USA

## Abstract

This retrospective cohort study evaluated the proportion of inpatients initiated and successfully discharged on fidaxomicin for *Clostridioides difficile* infection. Nearly, all patients (96.4%; 27/28) were able to obtain fidaxomicin to complete their treatment course, although there was variability in copays among patients with Medicare.

## Introduction/Background

Infectious Diseases Society of America (IDSA) guidelines identify fidaxomicin as the preferred treatment for both initial *Clostridioides difficile* infection (CDI) episode and first recurrence.^1^ A major barrier to widespread uptake is the potential high patient copay for fidaxomicin compared to alternative treatments; however, fidaxomicin might be becoming more financially accessible for patients. A recent study found that 93% of patients had a copay that was less than $50, with the highest copay ($1800) occurring in a Medicare patient.^2^ Although this single-center study suggested patient copays are decreasing, it is unclear if this is applicable to other hospitals. The objective of this study was to determine the proportion of inpatients at our academic center started on fidaxomicin for CDI who were successfully discharged with fidaxomicin to complete their CDI treatment. In addition, we evaluated fidaxomicin copay information and the effect of an outpatient transitions of care (TOC) pharmacy team at discharge.

## Methods

**Figure 1.**
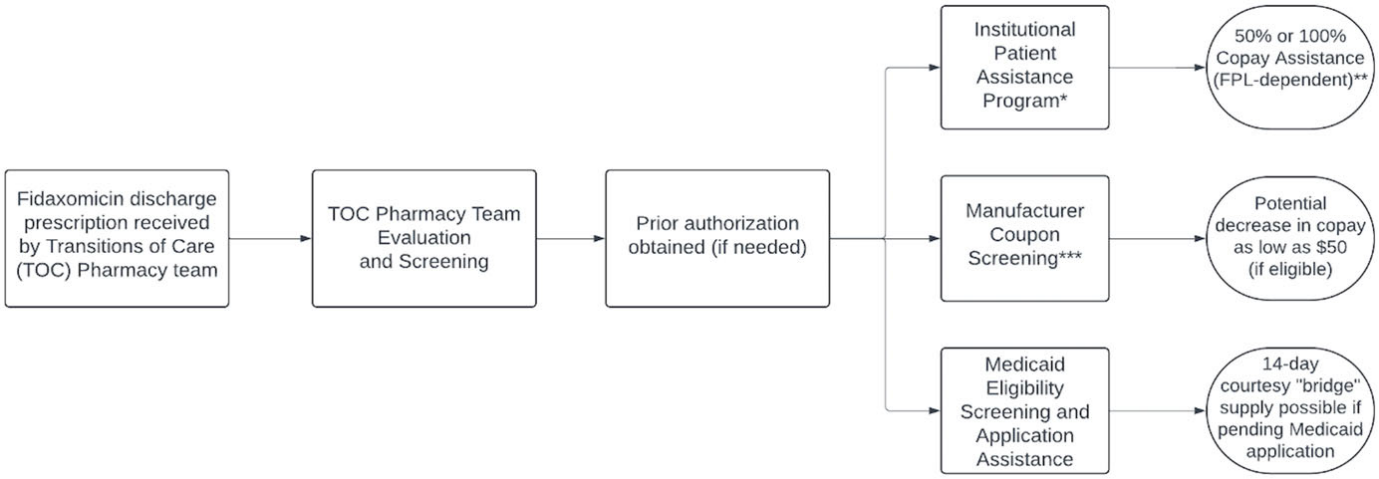
Fidaxomicin discharge prescription evaluation process by Transitions of Care (TOC) pharmacy team. *Screening for institutional assistance occurs if total copay ≥ $100 or if patient expresses inability to pay copay. **FPL = federal poverty level; 100% copay assistance if <300% FPL, 50% copay assistance if income between 301% and 500% FPL. ***Patients with Medicare or Medicaid insurance are not eligible for manufacturer coupons.

A retrospective chart review was completed for inpatients started on fidaxomicin at Beth Israel Deaconess Medical Center (Boston, MA) between January 1 2022 and June 30 2023. Patients were excluded if mortality occurred prior to finishing the course of fidaxomicin or if patients were continued on a previously prescribed outpatient course of fidaxomicin prior to their admission. Our institutional guidelines have oral vancomycin as preferred therapy for both initial episode and 1^st^ recurrence of CDI. Fidaxomicin is on inpatient formulary but requires approval by the Infectious Diseases (ID) consult teams or the Antimicrobial Stewardship Team (AST). The primary outcome was the proportion of patients successfully discharged on fidaxomicin, defined as confirmation of fidaxomicin on their discharge medication prescriptions or bedside delivery of medication if filled through our TOC pharmacy team. Secondary outcomes included initial patient copay, final patient out-of-pocket costs, and 30-day re-admission rates and mortality. Patient charts were reviewed for clinical, laboratory, and medication information. Additional pharmacy databases were reviewed by our TOC pharmacy team for insurance and copay information. Our TOC pharmacy team provides discharge prescription reviews, evaluation of financial barriers for high copay medications, and bedside medication delivery (Figure 1).

## Results

Forty-four patients were started on fidaxomicin during the study period that met inclusion criteria. Most patients were immunocompromised (24/44; 54.5%), had a history of prior CDI (24/44; 54.5%), and received ID consultation (26/44; 59%). 9% (4/44) of patients were switched from fidaxomicin to an alternative agent by the antimicrobial stewardship or primary team citing alignment with institutional guidelines; three patients were switched to oral vancomycin, and one patient was switched to PR vancomycin and IV metronidazole for fulminant CDI.

Of patients that were continued on fidaxomicin, 27% (12/44) completed their fidaxomicin course inpatient. For the 28 patients planned for fidaxomicin at discharge, 96.4% (27/28) were discharged successfully with fidaxomicin; 1 patient with Medicare insurance was not able to be discharged on fidaxomicin due to high co-pay ($1014). This patient declined TOC pharmacy screening and was discharged on oral vancomycin without CDI reoccurrence. Twenty-one patients were evaluated by the TOC pharmacy team (the remaining six did not have copay information available). Overall, the median copay for patients discharged on fidaxomicin was $30 with a mean copay of $243.88 (range $0–1384.95, SD = 408.87). When separated by insurance, 76% (16/21) had Medicare and 24% (5/21) had commercial insurance. The median copay for patients with Medicare was $30 and $50 for patients with commercial insurance. The mean copay for Medicare patients was $308.23 (range $0–1384.95, SD = 451.68) compared to $38 (range $0–75, SD = 35.81) for patients with commercial insurance. The number of patients did not allow adequate statistical power to detect a significant difference.

42.9% (9/21) of patients were classified in the “high copay” group (copay > $50) and 57.1% (12/21) were classified in the “low copay” group (copay < $50). Details of the “high copay” group (n = 9) and outcome of TOC evaluations are outlined in Table 1. Patients with Medicare insurance composed of all initial copays >$100. Even though 44% (4/9) patients were able to obtain financial assistance, two patients with Medicare paid final copays of $471.26 and $566.44. Of all patients (n = 44), one patient treated with fidaxomicin for 10 days was re-hospitalized secondary to CDI and was re-treated with an extended course of fidaxomicin. None of the patients that completed a course of fidaxomicin died within 30 days of discharge or fidaxomicin end date. 21% (5/24) of patients with a prior history of CDI received bezlotoxumab following discharge.

**Table 1.** Patients in the “High Copay” group with insurance and copay information (n = 9). Four patients (#6–9) had decreases in copay or paid no out-of-pocket costs after TOC evaluation. No patients were switched to alternative agents at discharge after final copays were determined

Patient number	Doses prescribed at discharge	Insurance	Initial patient copay ($)	TOC pharmacy intervention	Final patient out-of-pocket cost post-TOC intervention ($)
1	4	Commercial	65.00	None (copay under the minimum assistance)	65.00
2	13	Commercial	75.00	None (copay under the minimum assistance)	75.00
3	39 (taper regimen)	Medicare D	471.26	None (patient over income for assistance)	471.26
4	18	Medicare D	566.44	None (patient over income for assistance)	566.44
5	15	Medicare D	1265.88	None (dispensed through alternate pharmacy)^a^	Unknown
6	10	Medicare D	181.37	Institutional Assistance of 50%	90.69
7	7	Medicare D	547.76	Institutional Assistance of 100%	0
8	24 (taper regimen)	Medicare D	1384.95	Institutional Assistance of 100%	0
9	5	Medicare D	388.74	Qualified for Medicaid/Bridge supply provided^b^	0

a Confirmed that patient was able to obtain through Veterans Affairs pharmacy; however, final out-of-pocket cost was not able to be determined.

b TOC pharmacy team assisted with Medicaid application and provided courtesy “bridge” supply of 5 doses to the patient while application pending; allowed for $0 out-of-pocket cost at discharge.

## Discussion

Overall, nearly all patients (96.4%) needing fidaxomicin at discharge were able to obtain the medication to complete the course; however, the copay of the medication can be high for certain patients. At discharge, 43% of our patients had a copay higher than $50, notably higher than the 7% reported in a prior single center study, suggesting that economic barriers to fidaxomicin remain.^2^ Although the median copay for Medicare patients was lower than those with commercial insurance, there was a wider copay range, and Medicare patients had all copays greater than $100. This is likely due to fidaxomicin remaining in high copay tiers for many Medicare patients. A prior study found that fidaxomicin was on formulary for 84.1% of patients with Medicare; however, it was only tier 1 or tier 2 (lowest copay tiers) for 1.1% of patients.^3^ Notably, there is a manufacturer coupon that can lower the cost of fidaxomicin to as low as $50; however, Medicare and Medicaid patients are not eligible.

Our TOC pharmacy team assisted in decreasing the copay for some, but not all “high copay” patients. The benefit of TOC pharmacy teams on antimicrobial prescriptions has been previously shown to be beneficial in addressing pre-discharge financial barriers for antimicrobials in pediatric populations; however, no prior studies have included fidaxomicin.^4^ If an institution does have a TOC pharmacy team, close collaboration between clinical and antimicrobial stewardship teams is needed to ensure awareness of processes for evaluating the financial barriers of fidaxomicin (Figure 1).

Our study had several limitations. First, we did not evaluate outpatient fidaxomicin utilization or copays where the drug may be utilized more frequently. Additionally, we had a small inpatient cohort despite reviewing an 18-month period, reflecting institutional guidelines indicating oral vancomycin as the preferred CDI agent. Finally, this study was a single-center study in Massachusetts. Given the variability in insurance systems and tiers between states, there is likely wide variability in fidaxomicin copays. Our institution is fortunate to have a TOC team and medication assistance program described above, but results may not be generalizable to institutions without comparable resources to implement similar initiatives.

Our study found high rates of fidaxomicin discharges, although wide variability in copays remains possible. Primary clinical, infectious diseases, antimicrobial stewardship, and pharmacy teams should be aware of this possibility and collaborate to ensure processes are in place to help patients address high copays prior to discharge. Our TOC pharmacy team was able to reduce copay cost for nearly half of “high copay” individuals, demonstrating the importance of these teams, if available. Although a generic formulation of fidaxomicin was recently approved by the FDA in January 2024, the time line of widespread availability is unknown.^5^ In the interim, further strategies are needed to identify and address financial barriers for patients.
